# PM_2.5_ polluters disproportionately and systemically affect people of color in the United States

**DOI:** 10.1126/sciadv.abf4491

**Published:** 2021-04-28

**Authors:** Christopher W. Tessum, David A. Paolella, Sarah E. Chambliss, Joshua S. Apte, Jason D. Hill, Julian D. Marshall

**Affiliations:** 1 Department of Civil and Environmental Engineering, University of Illinois at Urbana-Champaign, Urbana, IL 61801, USA.; 2 Department of Civil and Environmental Engineering, University of Washington, Seattle, WA 98195, USA.; 3 Department of Civil, Architectural and Environmental Engineering, University of Texas at Austin, Austin, TX 78712, USA.; 4 Department of Civil and Environmental Engineering, University of California, Berkeley, Berkeley, CA 94720, USA.; 5 School of Public Health, University of California, Berkeley, Berkeley, CA 94720, USA.; 6 Department of Bioproducts and Biosystems Engineering, University of Minnesota, St. Paul, MN 55108, USA.

## Abstract

Racial-ethnic minorities in the United States are exposed to disproportionately high levels of ambient fine particulate air pollution (PM_2.5_), the largest environmental cause of human mortality. However, it is unknown which emission sources drive this disparity and whether differences exist by emission sector, geography, or demographics. Quantifying the PM_2.5_ exposure caused by each emitter type, we show that nearly all major emission categories—consistently across states, urban and rural areas, income levels, and exposure levels—contribute to the systemic PM_2.5_ exposure disparity experienced by people of color. We identify the most inequitable emission source types by state and city, thereby highlighting potential opportunities for addressing this persistent environmental inequity.

## Teaser

Nearly all major emission categories contribute to the systemic PM_2.5_ exposure disparity experienced by people of color.

## INTRODUCTION

Ambient fine particulate matter air pollution (PM_2.5_) is responsible for 85,000 to 200,000 excess deaths per year in the United States (*1*, *2*), with health effects observed even at concentrations below the current national standard of 12 μg m^−3^ (*3*–*5*). Racial-ethnic and socioeconomic disparities in air pollution exposure in the United States are well documented (*6*–*10*) and have persisted despite overall decreases in PM_2.5_ pollution (*11*–*13*).

Most evidence of exposure disparity relies on measured or empirically modeled ambient concentrations or on assessment of proximity to industrial or roadway emission sources (*6*, *10*, *12*–*20*). From the existing evidence, however, it is not possible to determine the relative contributions of different source types to racial-ethnic disparity in exposure to PM_2.5_. Here, we model anthropogenic sources of PM_2.5_ exposure resolved by race and ethnicity and show that nearly all major emission source sectors disproportionately affect people of color (POC).

We estimate exposure impacts for each emission source type on five racial-ethnic groups based on the U.S. Census: White (62% of the population), Black (12%), Hispanic (17%), Asian (5%), and POC (38%; see Materials and Methods for details). As a proxy for exposure to PM_2.5_, we calculate population-weighted average ambient PM_2.5_ concentrations for each race-ethnicity based on census-designated residential location.

We examine exposure disparity—the population-weighted concentration difference between each racial-ethnic group and the population average—in relative (percent) and absolute (μg m^−3^) terms. Sources with the highest relative disparity may yield the largest disparity mitigation per unit mass of emission reduction, whereas sources with the highest absolute disparity may have the greatest potential for overall disparity reduction.

## RESULTS

**Fig. 1:**
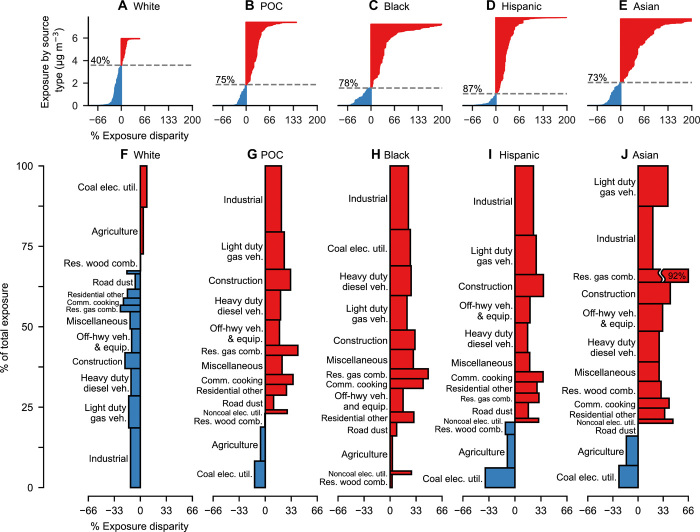
Source contributions to racial-ethnic disparity in PM_2.5_ exposure. (**A** to **E**) Individual source type (*n* = 5434 source types) contributions to exposure (*y* axis) and % exposure disparity (*x* axis, truncated at 200%, positive values are shaded red, negative values are shaded blue), with dashed lines denoting percent exposure caused by sources with positive exposure disparity. (**F** to **J**) Sources in (A) to (E) grouped into source sectors (*n* = 14 groups) and ranked vertically according to absolute exposure disparity, proportional to the area of each rectangle. As shown in (B), POC experience greater-than-average exposures from source types causing 75% of overall exposure. Source: data file S1, which also includes results for individual states and urban areas.

Results indicate that emission sources that disproportionately expose POC are pervasive throughout society. Estimated year 2014 total population average PM_2.5_ exposure from all domestic anthropogenic sources is 6.5 μg m^−3^ in the contiguous United States; exposures are higher than average for POC, Blacks, Hispanics, and Asians (7.4, 7.9, 7.2, and 7.7 μg m^−3^, respectively; Fig. 1, B to E) and lower than average for Whites (5.9 μg m^−3^; Fig. 1A). Whites are exposed to lower-than-average concentrations from emission source types causing 60% of overall exposure (Fig. 1A), with an overall relative exposure disparity of −8% (−0.55 μg m^−3^ absolute disparity) compared with the population average. Conversely, POC experience greater-than-average exposures from source types, causing 75% of overall exposure (Fig. 1B); their overall exposure disparity is 14% (0.90 μg m^−3^). Blacks are exposed to greater-than-average concentrations from source types contributing 78% of exposure (Fig. 1C), with an overall exposure disparity of 21% (1.36 μg m^−3^). Hispanics and Asians are disparately exposed to PM_2.5_ from 87 and 73% of sources, respectively, and experience 11% (0.72 μg m^−3^) and 18% (1.20 μg m^−3^) overall exposure disparities, respectively (Fig. 1, D and E).

Grouping the source types (Fig. 1, A to E) into 14 source sectors (Fig. 1, F to J) reveals that source types that disproportionately expose POC, Blacks, Hispanics, and Asians to higher-than-average concentrations are dominant in most sectors. Whites are exposed to lower-than-average concentrations from most emission sectors (Fig. 1F). Blacks are exposed to higher-than-average concentrations from all sectors (Fig. 1H).

Of the emission source sectors that cause the largest absolute disparities, four out of the top six source sectors are the same for POC, Blacks, Hispanics, and Asians: industry, light-duty gasoline vehicles, construction, and heavy-duty diesel vehicles (Fig. 1, G to J). Residential gas combustion and commercial cooking are among the largest sources of relative disparities for all four groups (e.g., 41 and 35%, respectively for POC; Fig. 1, G to J). The only sectors that affect Whites substantially more than average are coal electric generation and agriculture (8 and 4% relative disparity, respectively; Fig. 1F). Consistent with previous findings (*11*, *21*), we find that POC, Hispanics, and Asians are exposed to less PM_2.5_ from coal electric generators than average (−13%, −38%, and −18%, respectively), and Blacks are exposed to 18% more than average (Fig. 1H).

**Fig. 2:**
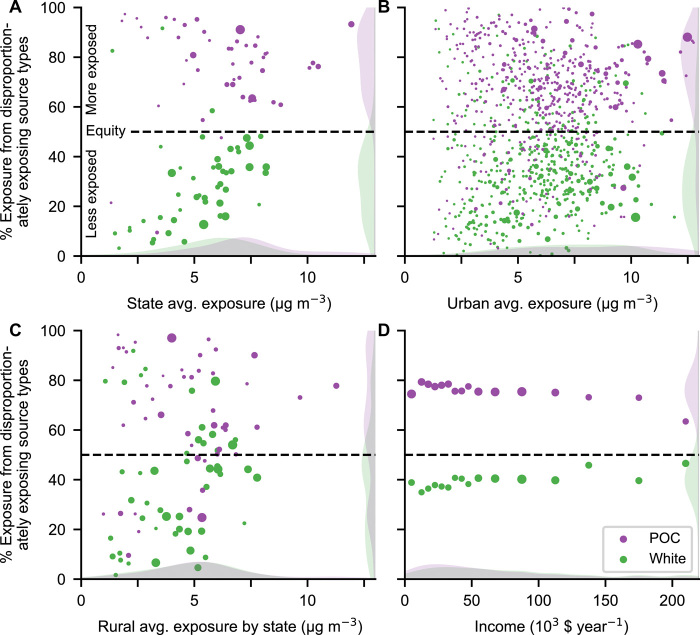
Percent of PM_2.5_ exposure caused by emission source types that disproportionately expose people of color (POC) and Whites. Data shown for (**A**) U.S. states (*n* = 48 states), (**B**) urbanized areas (*n* = 481 areas), (**C**) rural areas in each state (*n* = 48 states), and (**D**) income bins (*n* = 16 bins; last bin is >$200,000). Icon area is proportional to population; shaded areas are kernel density estimates. A *y* axis value of 50% would represent equity for that group (i.e., for the population-average exposure), meaning that half of their exposure comes from source types that disproportionately expose them and the other half is from source types that expose them less than average. Across geographies and levels of exposure (A to C), as well as incomes (D), most emission sources consistently result in higher exposures for POC and lower exposures for Whites. Source: data file S2.

**Fig. 3:**
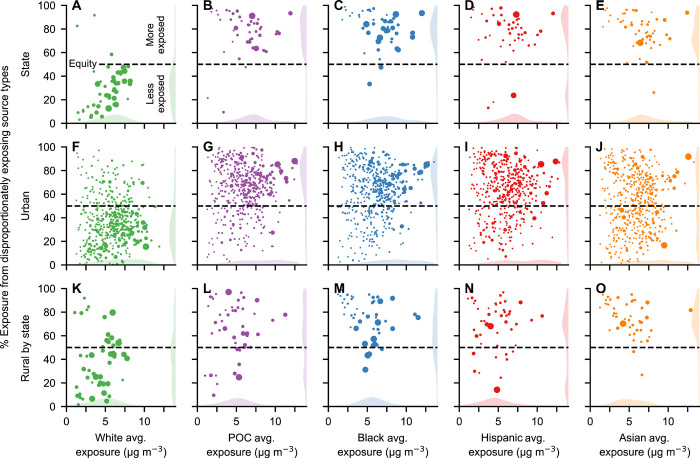
Percent of PM_2.5_ exposure caused by emission source types that disproportionately expose each racial-ethnic group by location and race-ethnicity. Icon area is proportional to population; shaded areas are kernel density estimates. This figure is analogous to Fig. 2 but with results for all five racial-ethnic groups. Source: data file S2.

Nationally, racial-ethnic exposure disparities are not caused by a small number of emission sources; instead, most source types and sectors result in higher-than-average exposures for POC and lower-than-average exposures for Whites (Fig. 1). By examining the percent of exposures caused by these disproportionately exposing emission source types for each group [for example, 40% for Whites (Fig. 1A) and 75% for POC (Fig. 1B) nationally], we find that this is also largely true within individual U.S. states, within individual urban and rural areas, across incomes, and across exposure levels (Figs. 2 and 3).

In 45 of the 48 states studied, disproportionately exposing sources cause the majority of POC exposure (Fig. 2A). In the (population-weighted) average state, 78% of exposure is caused by sources that disproportionately expose POC (White: 29%; Black: 77%; Hispanic: 73%; and Asian: 75%; Figs. 2A and 3, C to E; these average-state disparities differ from the national average disparities above because national averages include disparities occurring among states in addition to within states).

We observe the same effect within urban areas (Fig. 2B), with 73% of exposure in the (population-weighted) average urban area caused by sources that disproportionately expose POC (White: 31%; Black: 71%; Hispanic: 71%; and Asian: 56%; Figs. 2B and 3, H to J). There is a notable exception: Asians are less exposed than average in many urban areas in California with large Asian populations (data file S1; for example, Los Angeles, San Francisco, and San Jose). In the (population-weighted) average urban area outside California, 67% of Asian exposure is caused by source types that disproportionately expose Asians, compared with 56% when including California. Disparities also consistently occur in rural areas (defined here as the complement of urban areas), where a large proportion of exposure is caused by sources that disproportionately expose POC (White: 39%; POC: 62%; Black: 63%; Hispanic: 57%; and Asian: 74%; Figs. 2C and 3, M to O). However, disparities in rural areas are not as pronounced as in urban areas (Fig. 2, B and C).

Last, systemic disparity exists at all income levels. Consistent with a large body of evidence (*12*, *22*), we find that racial disparities are not simply a proxy for economic-based disparities. POC at every income level are disproportionately exposed by the majority of sources, with a population-weighted average across income bins of 76% of exposure caused by source types that disproportionately expose POC (Fig. 2D and fig. S1). Exposures vary more by race-ethnicity than by income: The difference in average exposure between POC and Whites is 2.4 times larger than the range in average POC exposure among income levels (data files S1 and S2).

## DISCUSSION

Our results come with caveats. First, we use emission amounts and locations, reduced complexity air quality modeling, and population counts that all contain previously quantified uncertainty (*11*, *23*; Supplementary Text). However, our core findings are consistent across states, urban and rural areas, and concentration levels, rendering it improbable that they are attributable to model or measurement bias. Second, because aggregate results are more robust than results for any single location, we recommend additional analysis incorporating local data and expertise before local actions are taken. Third, our results for states and for urban and rural areas reflect exposure to ambient PM_2.5_, including contributions from emission sources located outside the state, urban area, or rural area. This has implications for local authorities, who may not have jurisdiction over all sources of their exposure. Last, this analysis focuses on outdoor concentrations at locations of residence. Disparities in associated health impacts would also reflect racial-ethnic variability in mobility, microenvironment, outdoor-to-indoor concentration relationships, dose-response, access to health care, and baseline mortality and morbidity rates.

We have shown here that most emission source types—representing ~75% of exposure to PM_2.5_ in the United States—disproportionately affect racial-ethnic minorities. This phenomenon is systemic, holding for nearly all major sectors, as well as across states and urban and rural areas, income levels, and exposure levels. Industry, light-duty gasoline vehicles, construction, and heavy-duty diesel vehicles are often among the largest sources of disparity, but this can vary widely by source type and location. Because of a legacy of racist housing policy (fig. S2; supporting results) and other factors, racial-ethnic exposure disparities have persisted even as overall exposure has decreased (*11*–*13*). Targeting locally important sources for mitigation could be one way to counter this persistence. We hope the information provided here can help guide national, state, and local stakeholders to design policies to efficiently reduce environmental inequity.

## MATERIALS AND METHODS

We use a source-receptor matrix (*24*) created using the InMAP (*25*) air quality model to independently estimate concentrations in the contiguous United States resulting from anthropogenic emissions. We consider all 5434 source types [i.e., all U.S. Environmental Protection Agency (EPA) Source Classification Codes (SCCs) with nonzero emissions; we exclude the 8378 SCCs without emissions associated with them] in the 2014 EPA National Emissions Inventory (NEI) v1. County-level emissions are allocated to individual grid cells within the county using spatial surrogates. Emissions processing is described in further detail by Tessum *et al*. (*11*). To focus on impacts from modifiable factors, we do not investigate here emissions from biogenic, wildfire, or international sources. Exposure and health impacts resulting from these additional sources are quantified by Tessum *et al*. (*11*).

We investigate both primary (i.e., directly emitted) and secondary (i.e., formed in the atmosphere from other emissions) PM_2.5_. We model secondary PM_2.5_ formed from volatile organic compounds, oxides of nitrogen and sulfur (NO_x_ and SO_x_), and ammonia (NH_3_). We aggregate the 5434 SCCs (source “types”) into 14 source sectors (table S1), each accounting for >1% of total PM_2.5_ exposure. InMAP predicts concentrations at a spatial scale ranging from 48 km in areas with low population density to as fine as 1 km in urban centers; this intraurban spatial scale is necessary to resolve differences in exposure among demographic groups (*26*). The population-weighted average horizontal grid cell edge length is 10.8 km nationwide and 3.4 km in urban areas. Additional grid statistics can be found in table S2.

The source-receptor matrix relates emissions in any one location in a gridded spatial domain to InMAP-computed concentrations in all other locations. These relationships are generated with independent simulations of the air quality model for each of over 50,000 grid cells covering the contiguous United States for both ground-level and elevated sources.

Population-weighted average ambient concentrations, our measure of exposure, are calculated using a conventional approach to weighted averages. Specifically, we first multiply, for each grid cell, the population and the concentration. The sum of those values across all cells in the given spatial domain is then divided by the corresponding population to yield the population-weighted average concentration: PWA = Σ(*PC*)/Σ(*P*). Here, PWA is the population-weighted average, *P* is the population in a grid cell, *C* is the concentration in a grid cell, and the summations in the numerator and denominator are across all grid cells in the geography being studied (e.g., in a state, in the contiguous United States).

Population data by race-ethnicity are from the U.S. Census 2012–2016 American Community Survey (ACS) at Census Block Group level of spatial aggregation. We focus on the four largest race-ethnicity groups as determined by self-identification in the Census: Asian, Black or African American, Latino or Hispanic, and White. We aggregate these four population subgroups such that they are mutually exclusive: “Hispanic” including people of all races who identify as having Hispanic or Latino origin, and the other three groups (Asian, Black, and White) referring only to non-Latino/non-Hispanic persons. POC are defined herein as everyone except non-Latino/non-Hispanic Whites (i.e., individuals identifying as Hispanic plus non-Hispanic individuals identifying as Black or African American, American Indian or Alaska Native, Asian, Native Hawaiian and other pacific islander, some other race, or two or more races).

The 2012–2016 ACS provides income statistics by Census Tract, with 16 household income categories (lowest: “less than $10,000”; highest: “$200,000 or more”). We use the proportion of households in each income category to estimate population counts at the finest available level of race-ethnicity information: White and POC. Table S3 details the population distribution by income category.

To calculate exposure in individual urban areas, we use year 2018 urbanized area extents as defined by the U.S. Census (www.census.gov/geographies/mapping-files/time-series/geo/carto-boundary-file.html). We define “rural” as everywhere that is not within an urbanized area extent.

To calculate exposure by 1930s-era Home Owners’ Loan Corporation (HOLC) grades, we use historical maps digitized by the Mapping Inequality project (*27*). HOLC maps classify urban neighborhoods into four grades: A (green; “best”), B (blue; “still desirable”), C (yellow; “definitely declining”), and D (red; “hazardous”). For results shown in fig. S2, we define “% exposure from disproportionately exposing source types” as the percent of exposure that is caused by source types that expose residents of a given race-ethnicity currently living in an area with the given historical HOLC grade in a given city more than the overall average exposure of all residents of HOLC-graded areas in that city.

## SUPPLEMENTARY MATERIALS

Supplementary material for this article is available at http://advances.sciencemag.org/cgi/content/full/7/18/eabf4491/DC1
